# A method to accelerate computational efficiency by more than two orders of magnitude for Monte Carlo simulations of electron-solid interactions

**DOI:** 10.1038/s41598-024-64024-5

**Published:** 2024-06-17

**Authors:** Lin Shao

**Affiliations:** Accelerator Laboratory, Department of Nuclear Engineering, Texas A&M University, College Station, TX 77843 USA

**Keywords:** Monte Carlo method, Electron irradiation, Mott scattering, Free flying distances, Displacement creation, Electron ionization, Atomic and molecular collision processes, Computational methods

## Abstract

A method has been developed to increase computational efficiency in Monte Carlo simulations of electron transport and interactions in matter. The method serves as the computational engine for the open-source code AMCSET (Aggie Monte Carlo Simulations of Electron and Ion Transport). The key is to combine *n* consecutive neighboring free flying distances into groups. Within each group, both flying distance and Mott scattering angles are obtained using Monte Carlo sampling under an equal energy approximation. This reduces the number of integrations of the tabulated differential Mott scattering cross-section in scattering angle selection, i.e., from 1000 to 1 if *n* = 1000. The method increases efficiency by more than 100 times. At the same time, the calculation still guarantees accuracy in calculating electron trajectory, excitation/ionization energy deposition, elastic scattering energy deposition, and displacement creation. For demonstration, 10 MeV electron bombardments of pure Fe with *n* up to 1000 are used as examples. The method, due to the availability of tabulated scattering cross-sections, is applicable for targets of the entire elemental table up to Z = 118, and for electron energies up to 900 MeV.

## Introduction

Modeling of damage/radiation production caused by electron bombardment in matter are important for a wide range of applications, including materials imaging^1^, X-ray production^2^, radiation therapy^3^, materials modification^4–6^, and materials testing^7^. For applications such as scanning electron microscopy, electron bombardment energy is relatively low, i.e., < 50 keV. Other applications such as food irradiation or medical isotope production^8,9^, involve electron beam energy that is quite high, i.e., > 10 MeV. The variety of applications requires a simulation tool that is fast, accurate, and versatile for a wide range of electron beam energies.

An ideal simulator also requires comprehensive capability to simulate various by-products of electron-solid interactions, including secondary electrons, X-rays, and target atom displacements. Electron-solid interactions are commonly simulated using codes like MCNP^10^, GEANT4^11^, EGS5^12^, and CASINO^13^. While these codes are adept at predicting three-dimensional electron distributions, they fall short in providing three-dimensional defect distributions arising from damage cascades due to the creation of high-energy knock-on atoms (PKAs). In contrast, the binary Monte Carlo simulation code SRIM, which is extensively used, does not account for electron irradiation^14^. In the recently developed DEEPER code, electron transport was combined with a SRIM-like subroutine, which allows transferring the information of primary knock-on atoms into a full damage cascade simulator to obtain displacement production information^15^.

The computational efficiency, however, is a general issue for all electron simulations where sufficient accuracy is required. The relatively low efficiency is due to the complex nature of electron bombardment, which includes: (1) the specifics of both Mott scattering and electron screening effects in electron-nucleus scattering, which are unique to each element and require tabulated values, lacking analytical formulas for easy calculations; (2) the electron-nucleus scattering having a differential cross section that is significant at extremely small scattering angles. The latter necessitates a fine division of scattering angles to ensure accuracy. As will be discussed, this small scattering angle interval is the major issue limiting computational efficiency.

Here, a method that can significantly accelerate computational efficiency by orders of magnitude is presented. The key is to maintain a high number of scattering angle intervals to ensure accuracy, while reducing the frequency of Mott scattering cross-section integrations. This method, reported in the present study, serves as the computational engine for the open-source code AMCSET (Aggie Monte Carlo Simulations of Electron and Ion Transport), which will be released soon^16^. The code has the function of simulating both ion and electron transport, as well as point defect production. In the case of electron bombardment, the code proceeds into a subroutine to continuously simulate the displacement production if the energy transfer from electron-nuclei scattering exceeds the displacement threshold energies.

The rest of the article is organized as follows: First, we describe the general structure of the Monte Carlo simulation, with subsections covering simulation details of inelastic scattering, bremsstrahlung, and elastic scattering. Next, a special section is dedicated to detailing how to combine multiple neighboring flight distance calculations into equal-energetic groups. Lastly, the simulation of displacement production is explained as an extension of the simulation package.

## Modeling procedure

**Figure 1:**
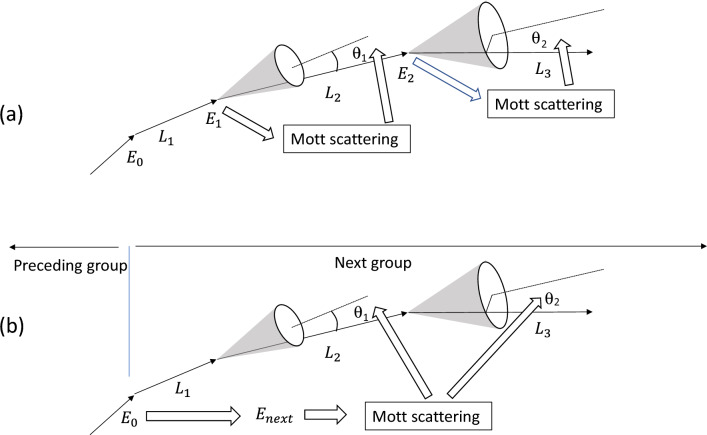
Schematics of scattering calculation used (**a**) in the traditional approaches and (**b**) in the present study.

In traditional Monte Carlo simulations, the scattering angle selection for each individual Mott scattering requires the assignment of a random number and an individual integration of the Mott scattering cross-section. As shown in Fig. 1a, the traditional approach requires the updated energy, ${E}_{i}$, after each flying distance, ${L}_{i}$, and evaluation of Mott scattering for determining ${\theta }_{i}$. This is very time-consuming and computationally costly, since each element has its own tabulated scattering parameters, and the integration requires an extremely small angle interval.

In the present study, combining neighboring free flying distances into one group is proposed, and a single integration is used to ascertain multiple scattering angles from multiple random numbers for multiple free flying distances within the group. As shown in Fig. 1b, the electron scattering is divided into groups. The ending energy of the preceding group is used as an input to estimate the average energy for the next group. Only one integration of the Mott differential scattering cross section is needed to create scattering angles for each Mott scattering within the next group. This approach leads to a marked improvement in efficiency, substantially reducing computation time by orders of magnitude. The combination of free flying distances into groups does not reduce the number of collision steps. The total number of Mott scatterings remains the same as in the traditional approach, as indicated in Fig. 1a, b. As an example, in the present study, a combination of 1000 free flying distances (n = 1000) is demonstrated. The key is to predict 1000 various Mott scattering events within the group by integrating the Mott differential scattering cross section once under an equal energy approximation. This process requires the selection of 1000 random numbers, ordering these numbers from low to high, finding the corresponding Mott scattering, and then reordering the scattering angles randomly within the group. For each collision within a group, its free flying distance is separately selected using another set of random numbers, and energy after each collision is updated to reflect energy losses, including both continuous energy loss via inelastic scattering and discrete energy loss via Mott scattering.

Our method is applicable for electron irradiation but cannot be applied to other types of particle bombardments such as ion bombardment. This is due to the uniqueness of Mott scattering, which determines the trajectory of electrons, but whose energy loss is almost negligible compared to inelastic energy loss. This feature is important, allowing neighboring Mott scatterings to be evaluated under an approximately constant energy. If Mott scattering were a major energy loss mechanism, Mott scattering within the same group would become sensitive to its preceding scattering events, necessitating that scatterings be evaluated sequentially, one after another, as in the case of ion bombardment. Additionally, Mott scattering is not highly sensitive to energy changes, which allows for a constant energy approximation within a reasonably selected grouping size. Additional justification and details of the proposed method will be further explained in Sect. "Modeling procedure".

The simulations in the present study consider ionization/excitation, bremsstrahlung, and Mott scattering. Ionization/excitation and bremsstrahlung are often treated as continuous energy loss, while Mott scattering is typically treated as discrete energy loss. The statistical fluctuations of the energy loss from excitation and ionization are ignored in the present study. To be precise, all interaction types can be treated as discrete energy losses and modeled as individual scattering events. In practice, free flying distances are introduced to simplify calculations by avoiding the need to compute each individual interaction event. A free flying distance represents the separation distance between two consecutive Mott scatterings. Within each free flying distance, energy loss due to excitation/ionization and X-ray production is calculated as the product of the flying distance and the corresponding stopping power. At the end of each free flight distance, a Monte Carlo approach is used to sample the deflection angle of Mott scattering. Below, the simulation details of each interaction mechanism are separately discussed.

### Energy loss due to inelastic *electron*–*electron* scattering

For electrons with kinetic energies below 10 keV, corresponding to velocities significantly lower than the speed of light, the energy loss is calculated by a modified Bethe equation given below^17–19^:

1 $$\left( {\frac{dE}{{ds}}} \right)_{ionization} = 7.85 \times 10^{4} \frac{N}{{N_{a} }}\frac{Z}{{E_{k} }}\ln \left( {\frac{{1.166E_{k} }}{{J^{*} }}} \right) {\text{keV/cm}}$$

where.

$N:$ the target atomic density in the unit of atoms/cm^3^.

${N}_{a}$: the Avogadro’s number (${N}_{a}=6.02214076\times {10}^{23}$).

$Z:$ The atomic number of target atoms.

${E}_{k}:$ The kinetic energy of electron (in the unit of keV).

J*: The modified mean ionization potential (in the unit of keV).

*s*: the flying distance.

The modified mean ionization potential, J*, is calculated by^18^:

$$J* = \frac{J}{{1 + k\frac{J}{{E_{{\text{k}}} }}}}$$

$$J=\left\{\begin{array}{c}0.0115Z \left(Z<13\right)\\ 0.00976Z+0.0585{Z}^{-0.19} \left(Z\ge 13\right)\end{array}\right.$$

2 $$k = 0.734Z^{0.037}$$

For high-speed electrons, a relativistic form of Bethe equation is used^20^:

3 $$\left( {\frac{dE}{{ds}}} \right)_{ionization} = 153.55 \times \frac{N}{{N_{a} }}\frac{Z}{{\beta^{2} }}\left[ {\ln \left( {\frac{{m_{e} c^{2} \beta^{2} E_{k} }}{{2J^{2} \left( {1 - \beta^{2} } \right)}}} \right) - \ln 2\left( {2\sqrt {1 - \beta^{2} } - 1 + \beta^{2} } \right) + } \right.\left. {\left( {1 - \beta^{2} } \right) + \frac{1}{8}\left( {1 - \sqrt {1 - \beta^{2} } } \right)^{2} } \right] {\text{keV/cm}}$$

where ${m}_{e}{c}^{2}$ is the rest mass energy of an electron in the unit of keV (${m}_{e}{c}^{2}=511 keV). \beta$ is the speed of electrons normalized by the speed of light, *β* = *ν/c.*

### Energy loss due to Bremsstrahlung

Bremsstrahlung is electromagnetic radiation which is significant when electron energies are significantly high. The corresponding stopping power of electrons, measured in units of keV/cm, contributed by the Bremsstrahlung radiation, is calculated by^21^:

4 $$\left( {\frac{dE}{{ds}}} \right)_{Brems.} \approx 1.4 \times 10^{ - 4} \frac{N}{{N_{a} }}Z\left( {Z + 1} \right)\left( {E_{k} + m_{e} c^{2} } \right)\begin{array}{*{20}c} {\left[ {4ln\left( {\frac{{2\left( {E_{k} + m_{e} c^{2} } \right)}}{{m_{e} c^{2} }}} \right) - \frac{4}{3}} \right]} & {} \\ \end{array} {\text{keV/cm}}$$

### Mott scattering

For describing electron-nucleus scattering, the common approach is to modify the Rutherford scattering cross section with a Mott correction. The Rutherford scattering cross section is calculated by treating the target nucleus as an isolated point charge, without considering the presence of atomic electrons. The more precise Mott cross sections consider (1) the effect of electron spins during scattering, in addition to the Coulombic force, (2) relativistic effects for high-energy electrons, and (3) the electron screening effect. The latter is due to the presence of atomic electrons, which creates a shielding effect, reducing the effective charge of the nucleus to less than its atomic number.

Considering the aforementioned effects, the differential scattering cross section for electron-nucleus scattering can be described as follows^22^:

5 $$\frac{d{\sigma }_{Mott}}{d\Omega }={R}_{Mott}\times \frac{d{\sigma }_{Ruth}}{d\Omega }\times {\left[1-{F}_{e}\left(q\right)\right]}^{2}$$

where $\frac{d{\sigma }_{Ruth}}{d\Omega }$ is the Rutherford scattering cross section. ${R}_{Mott}$ is the Mott ratio which is for the correction after considering the electron spin effect but not electron-screening effect. The term ${\left[1-{F}_{e}\left(q\right)\right]}^{2}$ is for the correction considering the electron screening effect. The parameter $q$ is a function of electron velocity and Mott scattering angle, and is to be further explained shortly.

An approximation of the Rutherford scattering cross section is given by^23^:

6 $$\frac{d{\sigma }_{Ruth}}{d\Omega }={\left(Z\times {r}_{e}\right)}^{2}\times \left(\frac{1-{\beta }^{2}}{{\beta }^{4}}\right)\times \frac{1}{{\left(1-\mathit{cos}\theta \right)}^{2}}$$

where *θ* is the scattering angle of electrons, and *r*_*e*_ is the classic radius of electron (*r*_*e*_ = 2.817938 × 10^-13^ cm).

The Mott treatment is based on a method from Wenzel^24^, originally used to deal with incident and scattered waves on a point-like nucleus. After considering the spin effect, the obtained screening-free Mott differential cross sections are formulated as two conditionally convergent infinite series in terms of Legendre expansions^22^. A simple analytic expression of *R*_*Mott*_ is available but it is limited to low Z materials^25^. For wider energy regions and more general target atoms, a fitting formula can be used with tabulated parameters. One generally accepted format is expressed in a polynomial expression^26^:

7 $${R}_{Mott}={\sum }_{j=0}^{4}{\alpha }_{j}\left(Z,\beta \right)(1-\mathit{cos}\theta {)}^\frac{j}{2}$$

8 $${\alpha }_{j}\left(Z,\beta \right)={\sum }_{k=1}^{6}{b}_{k,j}(Z)(\beta -\overline{\beta }{)}^{k-1}$$

$\overline{\beta }$ = 0.7181287 corresponds to the average velocity of electrons at 0.7181287c within the energy range of 1 keV to 900 MeV. ${\alpha }_{j}$ and ${b}_{k,j}$ are fitting parameters which were originally proposed by Lijian et al. for elements up to Z = 90^26^. Boschini et al. provided an updated table for elements up to Z = 118^22^.

The term [1-Fe(q)]^2^, which accounts for screening effects, varies depending on the proximity of the electron to a target nucleus and the specific electron configurations of the target atoms. Consequently, it is dependent on both energy and the atomic number of the target atoms. If the Dirac-Hartree–Fock-Slater screening function is used, ${F}_{e}\left(q\right)$ can be expressed by^27^:

9 $${F}_{e}\left(q\right)=\sum_{i=1}^{3}{A}_{i}\frac{{\left[\frac{h{a}_{i}}{2\pi }\right]}^{2}}{{\left[\frac{h{\alpha }_{i}}{2\pi }\right]}^{2}+{q}^{2}}$$

where *h* is the Plank constant, *A*_*i*_ and *α*_*i*_ are parameters to describe the screening function, and *q* is the momentum transfer, calculated by^27^:

10 $$q=2\times \beta \times \gamma \times {m}_{e}\times c\times \mathit{sin}\frac{\theta }{2}$$

#### Determination of scattering angle under Mott scattering

Combining Eqs. (5–10), the total cross section for electron-nucleus scattering is calculated by the integration:

11 $${\sigma }_{T}={\int }_{0}^{\pi }\frac{d{\sigma }_{Mott}}{d\Omega }\cdot 2\pi \cdot sin\theta \cdot d\theta$$

Next, a random number ${R}_{\theta }$, which must be in the range of 0 to 1, is used to select the scattering angle at the end of each free flying distance, calculated by

12 $${R}_{\theta }=\frac{{\sigma }_{Mott}\left(\theta \right)}{{\sigma }_{T}}=\underset{0}{\overset{\theta }{\int }}\frac{d{\sigma }_{Mott}\left({\theta }{\prime}\right)}{d\Omega }\cdot2\pi \cdot sin\theta {\prime}\cdot d\theta {\prime}/{\sigma }_{T}$$

where ${\sigma }_{Mott}\left(\theta \right)$ represents the integration of the differential cross section up to the angle $\theta$. In practice, the maximum allowable scattering angle, $\pi ,$ is divided into *m* intervals. The scattering angle θ falls in the region between ${\theta }_{i}$ and ${\theta }_{i+1}$ if ${R}_{\theta }$ satisfies

13 $$\frac{{\sum }_{{i}{\prime}=1}^{{i}{\prime}=i} \frac{d{\sigma }_{Mott}\left({\theta }_{{i}{\prime}}\right)}{d\Omega }\cdot 2\pi \cdot sin{\theta }_{{i}{\prime}}\Delta {\theta }_{{i}{\prime}}}{{\sigma }_{T}}<{R}_{\theta }\le \frac{{\sum }_{{i}{\prime}=1}^{{i}{\prime}=i+1} \frac{{d\sigma }_{Mott}\left({\theta }_{{i}{\prime}}\right)}{d\Omega }\cdot 2\pi \cdot sin{\theta }_{{i}{\prime}}\Delta {\theta }_{{i}{\prime}}}{{\sigma }_{T}}$$

where $\Delta {\theta }_{i}$ is the interval width for ${\theta }_{i}$. Since $\frac{d{\sigma }_{Mott}\left(\theta \right)}{d\Omega }$ is very high at small angles, ${\theta }_{i}$ should be unevenly distributed, favoring a higher density at small angles. One such choice is:

14 $${\theta }_{i}=\pi \frac{1-{10}^\frac{i}{m}}{1-10}$$

where $m$ is the number of angle intervals used in the cross-section calculation, $i$ is an integer in the range of $1\le i\le m-1$. $\Delta {\theta }_{i}$ for $2\le i\le m-2$ is calculated as the distance between $\frac{{\theta }_{i-1}+{\theta }_{i}}{2}$ and $\frac{{\theta }_{i}+{\theta }_{i+1}}{2}$. At two boundaries, $\Delta {\theta }_{1}$ is the distance between 0 to $\frac{{\theta }_{1}+{\theta }_{2}}{2}$, and $\Delta {\theta }_{m-1}$ is the distance between $\frac{{\theta }_{m-2}+{\theta }_{m-1}}{2}$ and $\pi$.

Once *θ* is determined to be in the region between ${\theta }_{i}$ and ${\theta }_{i+1}$ through Eq. (13), its exact value needs to be more precisely determined based on the distance from the upper integration boundary $\frac{{\theta }_{i+1}+{\theta }_{i+2}}{2}$. It is important to note that this boundary value is slightly larger than ${\theta }_{i+1}$ due to the selection of $\Delta {\theta }_{i+1}$. The calculation assumes ${\sigma }_{Mott}\left(\theta \right)$ and $\theta$ around ${\theta }_{i+1}$ follow the same linear proportionality. For $2\le i\le m-2, \theta$ is calculated by:

15 $$\theta =\frac{{\theta }_{i+1}+{\theta }_{i+2}}{2}-\left[\frac{{\sum }_{{i}{\prime}=1}^{{i}{\prime}=i+1}\frac{{\sigma }_{Mott}\left({\theta }_{i{\prime}}\right)}{d\Omega }\cdot 2\pi \cdot sin{\theta }_{i{\prime}}\Delta {\theta }_{i{\prime}}}{{\sigma }_{T}}-{R}_{\theta }\right]\cdot \frac{\Delta {\theta }_{i+1}}{\frac{{\sigma }_{Mott}\left({\theta }_{i+1}\right)}{d\Omega }\cdot 2\pi \cdot sin{\theta }_{i+1}\Delta {\theta }_{i+1}\cdot \frac{1}{{\sigma }_{T}}}$$

With

$$\Delta {\theta }_{i+1}=\frac{{\theta }_{i+2}-{\theta }_{i}}{2}$$

Due to the high likelihood of ${R}_{\theta }$ being less than $\frac{{\sigma }_{Mott}\left({\theta }_{1}\right)}{{\sigma }_{T}}$ and the specific starting boundary selection for $\Delta {\theta }_{1}$, the selection of θ between 0 and ${\theta }_{1}$ is calculated by:

16 $$\theta =\frac{{\theta }_{1}+{\theta }_{2}}{2}-\left[\frac{\frac{{\sigma }_{Mott}\left({\theta }_{1}\right)}{d\Omega }\cdot 2\pi \cdot sin{\theta }_{1}\Delta {\theta }_{1}}{{\sigma }_{T}}-{R}_{\theta }\right]\cdot \frac{\Delta {\theta }_{1}}{\frac{{\sigma }_{Mott}\left({\theta }_{1}\right)}{d\Omega }\cdot 2\pi \cdot sin{\theta }_{1}\Delta {\theta }_{1}\cdot \frac{1}{{\sigma }_{T}}}$$

with

$$\Delta {\theta }_{1}=\frac{{\theta }_{1}+{\theta }_{2}}{2}$$

If θ falls into the last interval region, where $\frac{{\sigma }_{Mott}\left({\theta }_{m-2}\right)}{{\sigma }_{T}}<{R}_{\theta }\le 1$, then, due to the specific ending boundary selection, θ is calculated by:

17 $$\theta =\pi -\left[1-{R}_{\theta }\right]\cdot \frac{\Delta {\theta }_{m-1}}{\frac{{\sigma }_{Mott}\left({\theta }_{m-1}\right)}{d\Omega }\cdot2\pi \cdot sin{\theta }_{m-1}\Delta {\theta }_{m-1}\cdot \frac{1}{{\sigma }_{T}}}$$

with

$$\Delta {\theta }_{m-1}= \pi -\frac{{\theta }_{m-2}+{\theta }_{m-1}}{2}$$

The selection of $\theta$ for each Mott scattering represents the most time-consuming step in the entire Monte Carlo simulation. High accuracy necessitates calculating Eq. (12) with Δθ at an extremely small value (Δθ $\ll$ 1°). This requirement is due to the sharp changes of $\frac{d{\sigma }_{Mott}\left({\theta }{\prime}\right)}{d\Omega }$ at very small angles.

**Figure 2:**
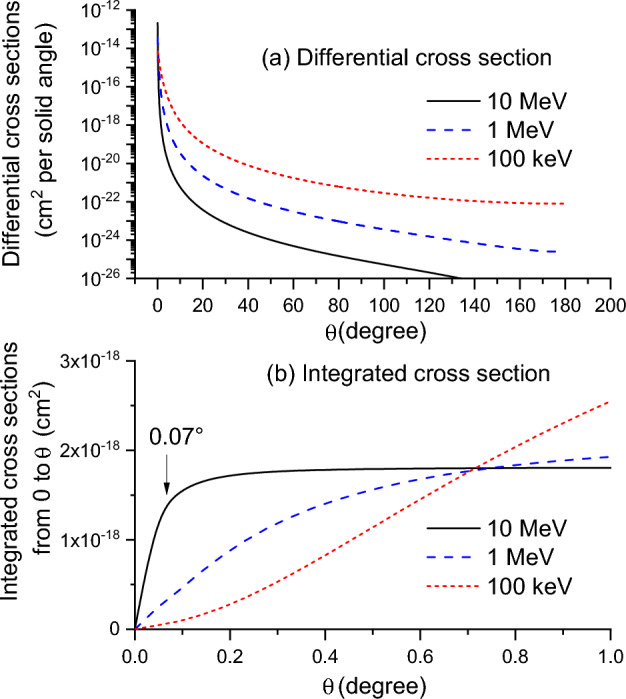
(**a**) Differential cross sections as a function of scattering angle θ in Fe and (**b**) integrated cross sections from angle 0° to θ, for 10 MeV (dark solid line), 1 MeV (blue dashed line), and 100 keV (red dotted line) electrons.

In Fig. 2a, $\frac{d{\sigma }_{Mott}\left(\theta \right)}{d\Omega }$ of electron scattering by Fe at energies of 10 MeV, 1 MeV, and 100 keV is plotted. For 10 MeV electrons, when angles change from 0.1° degree to 1°, the differential cross sections are reduced by four orders of magnitude. This sharp reduction at extremely small angles results in a quick rise of ${\sigma }_{Mott}\left(\theta \right)$ at small angles, as shown in Fig. 2b for $\theta <1^\circ$. For 10 MeV electrons, ${\sigma }_{Mott}\left(\theta \right)$ quickly increases and saturates around a value of ~ 1.8 × 10^–18^ cm^2^ at $\theta$ > 0.2°. Normalized by the saturated value at large $\theta$, $\frac{{\sigma }_{Mott}\left(\theta \right)}{{\sigma }_{Mott}\left(\theta \gg 1\right)}$ reaches about 0.5 at $\theta =0.035^\circ$ and about 0.6 at $\theta =0.042^\circ$. This means that, to differentiate random numbers 0.5 and 0.6, the selection procedure requires $\theta$ to be differentiable at a resolution of 0.007 ^∘^. Equation (15) allows the extraction of $\theta$ even when it is less than the first interval width $\Delta {\theta }_{1}$*.* However, this equation is based on the assumption of linear proportionality within $\Delta {\theta }_{1}$. Therefore, $\Delta {\theta }_{1}$ cannot be wider than the critical $\theta$ value where the ${\sigma }_{Mott}\left(\theta \right)$ value begins to deviate from this linear proportionality. The arrow in Fig. 2b marks the position of linear proportionality limit, which is 0.07 ^∘^. Based on Eq. (14) and the formula $\Delta {\theta }_{1}=\frac{1}{2}\left({\theta }_{1}+{\theta }_{2}\right),$ it can be determined that interval number *n* cannot be less than 1000. If an evenly divided interval is used, as opposed to the method in Eq. (14), the interval number increases to 2600.

This results in an incredibly large number of angle intervals for Monte Carlo simulations, significantly increasing the computation time. For lower energies, such as 1 MeV and 100 keV, the changes in $\frac{d{\sigma }_{Mott}\left({\theta }{\prime}\right)}{d\Omega }$ at small $\theta$ are less pronounced. Additionally, the saturation values of ${\sigma }_{Mott}\left(\theta \right)$ increases, and saturation begins at larger $\theta$ values. These factors relax the requirement of small $\Delta \theta$ compared to the 10 MeV case. However, the number of angle intervals required remains significantly large, thereby slowing down the computation. For the 100 keV case, the ${\sigma }_{Mott}\left(\theta \right)$ profile shows a shape different from both the 1 MeV and 10 MeV cases. Compared with 1 MeV, its proportional limit shifts to $\theta \approx 0.012^\circ$. Still, it requires a large *n*.

#### Determination of free flying distances under Mott scattering

The free flying distance of electrons must conform to Poisson distributions. Accordingly, the selection of the free flying distance, denoted as *L*, in Monte Carlo sampling for each individual Mott scattering event is calculated by:

18 $$L=-\frac{ln\left({R}_{L}\right)}{N\cdot {\sigma }_{T}}$$

where ${\sigma }_{T}$ is the total Mott scattering cross section, as obtained from Eq. (11). ${R}_{L}$ is a random number created to determine free flying distances. Note that ${R}_{L}$ and ${R}_{\theta }$ are different random numbers used for different purposes.

**Figure 3:**
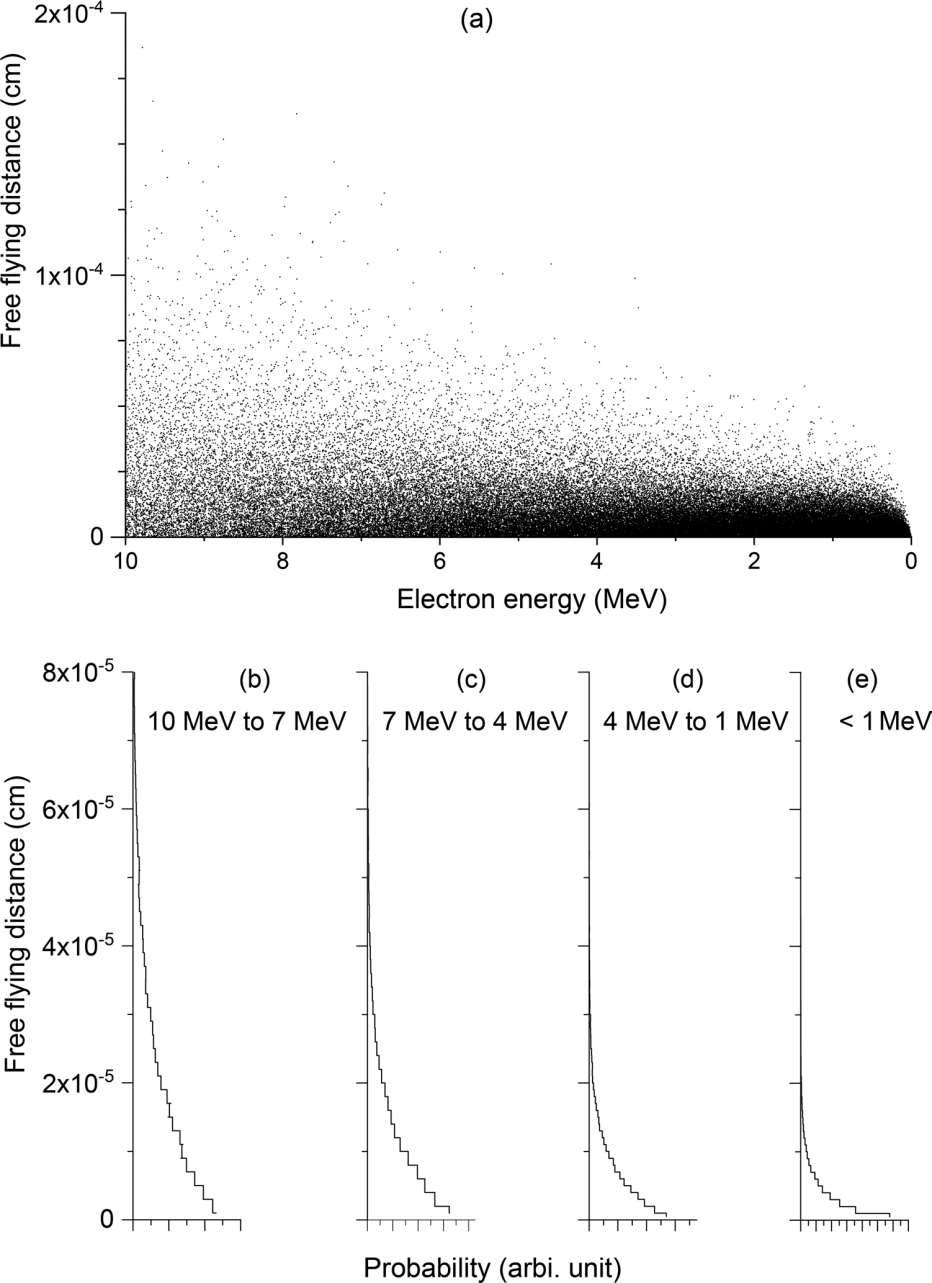
(**a**) The selection of free flying distance as a function of local electron energy for the simulation of one single electron bombarding pure Fe, at an incident energy of 10 MeV. (**b**–**e**) the statistics analysis of $\text{L}$ selection for the electron energies in the range of 10–7 MeV, 7–4 MeV, 4–1 MeV, and < 1 MeV. The number of angle intervals is 1000.

With $N=8.482\times {10}^{22}$ atoms/cm^3^ for pure Fe, $L$ is calculated for 10 MeV electron bombardment using Eq. (18). Figure 3a plots the distributions of $L$ selections along the trajectory of one 10 MeV electron. Figures 3b-e plot the statistical distributions of $L$ for electron energy ranges of 10–7 MeV, 7–4 MeV, 4–1 MeV, and less than 1 MeV, respectively. The distributions of $L$ values become narrower and the mean free flying distance gradually reduces to short distances as the local electron energies decrease. This trend is attributed to the energy dependence of Mott cross section. The total cross section, ${\sigma }_{T},$ increases with decreasing electron energy. The probability distribution profiles of Figs. 3b-e follow the expected Poisson distribution functions.

Given that the total flying distance for a 10 MeV electron is approximately 0.8 cm (to be discussed shortly), the total number of $L$ selections required for a single electron bombardment simulation is significant. The total number of free distance selections in Fig. 3a, for a single electron bombardment is approximately 90,000. For any meaningful Monte Carlo simulations, more than 1000 electron bombardments are likely required for reliable statistics. Additionally, considering that each free flying distance involves the integration of Mott scattering cross section with the number of angle intervals at 1000 or higher, the simulation becomes very time-consuming. This necessitates an innovative approach, which will be further explained in the next section.

#### Energy loss due to Mott scattering

To calculate the energy loss after each Mott scattering, the following general formula is used^28^:

19 $$E_{t} = \frac{{\left[ {\left( {E_{k} + m_{e} c^{2} } \right)\sin^{2} \theta + Mc^{2} \left( {1 - \cos \theta } \right)} \right]E_{k} \left( {E_{k} + 2m_{e} c^{2} } \right)}}{{\left( {E_{k} + Mc^{2} } \right)^{2} - E_{k} \left( {E_{k} + 2m_{e} c^{2} } \right)\cos^{2} \theta }}$$

where *M* is the mass of the target atom. *E*_*k*_ = *m*_*e*_*c*^*2*^((*1-β*^*2*^)^*-1/2*^*–*1).

### Proposed new method to increase compactional efficiency

The key approach to improving computational efficiency is to reduce the number of times Mott scattering cross sections are integrated. As will be explained, this can be achieved by combining neighboring free flying distances into groups, assuming that each individual Mott scattering within the group can be simulated using the same electron energy. If so, only one integration of Mott scattering is needed for one group to determine all scattering angles. Below, we first provide justification for why such an approach is valid and appropriate. Then, the methodology of grouping free flying distances and calculating scattering angles within the group is introduced. Lastly, a comparison is made between traditional approaches and the proposed new methods to show the efficiency and accuracy of the new method.

**Figure 4:**
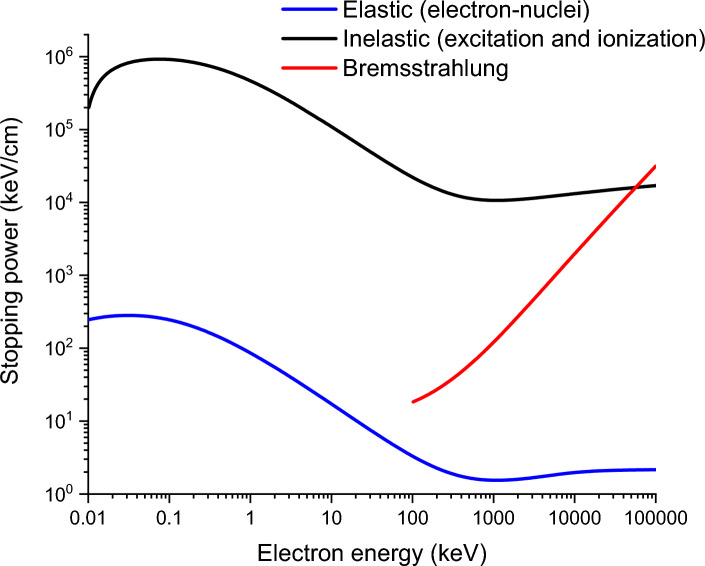
The stopping power (energy loss per unit flying distance) contributed by inelastic scattering (excitation and ionization), bremsstrahlung, and elastic scattering (Mott scattering) in Fe.

The equal-energy combination of multiple free flying distances is based on the fact that Mott scattering is most significant in changing the trajectories of electrons but much less significant in causing energy loss. Figure 4 compares the stopping powers contributed by elastic scattering (Mott scattering), inelastic scattering (excitation and ionization), and bremsstrahlung, using electron bombardments in Fe as an example. The energy loss due to excitation and ionization is the most significant. The energy loss due to Mott scattering is systematically lower than that due to excitation/ionization by at least three orders of magnitude for the entire energy range. Bremsstrahlung becomes more significant than excitation and ionization at electron energies exceeding ~ 60 MeV.

The insignificance of Mott scattering in energy transfer suggests that the local electron energy along their flying trajectory is not affected by the selection of Mott scattering angles. In other words, Mott scattering can be repeatedly evaluated assuming the same electron energy over a certain flying distance. As illustrated by Fig. 3b–e, the average free flying distance is on the order of 1 × 10^–6^ cm. Hence, the equal energy assumption can be applied to, for instance, 1000 combined free flying distances.

**Figure 5:**
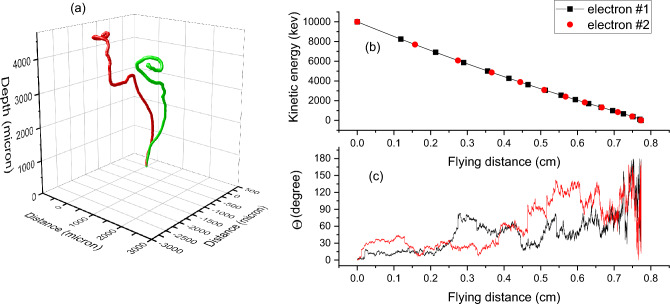
(**a**) 3D trajectories of two electrons in Fe, both at an incident energy of 10 MeV; (**b**) local kinetic energies as a function of flying distances for both electrons; (**c**) local θ values with respect to the z-axis as a function of flying distance for both electrons. Each line in (b) contains about 90,000 points (free flying distances). The number of angle intervals is 1000.

One consequence of the almost negligible energy loss in Mott scattering (in comparison with the energy loss due to excitation and ionization, as shown in Fig. 4) is that the accumulated flying distances, integrated over the entire trajectory, are roughly the same for all electrons (as long as they are stopped inside the substrate). Figure 5a shows the trajectories of two 10 MeV electrons. Both exhibit large trajectory changes. Figure 5b plots the kinetic changes of both electrons as a function of flying distances. Black symbols and red symbols are used to differentiate the two electrons. Both electrons travel roughly the same distance of about 0.78 cm, although both have very large deflection histories as shown by the $\Theta$ values in Fig. 5c. $\Theta$ is the angle with respect to the z-axis. The same flying distances for both electrons are a consequence of the fact that kinetic energy loss is insensitive to Mott scattering. Even though individual scattering events show large differences in scattering direction changes and energy transfer, overall, Mott scattering does not cause significant differences in the total flying distances.

Another significant aspect of the results from Fig. 5b is the consistency in the slope, which appears to be similar throughout the entire flying journey. It's important to note that this slope represents the stopping power ($\frac{dE}{ds}$). A consistent slope indicates that the stopping power for neighboring flight distance groups is roughly the same. This provides justification for why each Mott scattering event within a group can be calculated using the same energy level—specifically, the midpoint energy within the group. Although the actual energy within the group changes due to accumulated energy loss, such gradual changes do not significantly affect the stopping power. Hence, it is acceptable to use a singular stopping power value (a consequence of the equal energy approximation) to calculate the stopping loss across the entire group.

Our proposed method includes the following steps:

1. Define an integer number *n* as the size of the group, which is the number of flying distances included in each group. For the present study, *n* is up to 1000.
2. Produce *n* random numbers for each group. Each random number is in the range (0,1).
3. Sort the random numbers in their original sequence from smallest to largest: ${R}_{\theta }(i)<{R}_{\theta }(i+1)$ for $i$ from 1 to $n-1$.
4. Calculate ${\theta }_{i}$ for each random number from low to high: ${R}_{\theta }(i)=\frac{{\sigma }_{Mott}\left({\theta }_{i}\right)}{{\sigma }_{T}}$. Once the entire set of *θ*_i_ values is determined, they are randomly reordered.
5. Calculate the energy transfer for each ${\theta }_{i}$ value.
6. For each kinetic energy transfer obtained using the individual scattering angle within a group, determine whether the transferred kinetic energy exceeds the threshold for displacement creation. If one or more displacements are created, transfer the knock-on information to a subroutine for subsequent damage cascade calculation (details to be discussed).
7. Calculate the total kinetic energy loss from Mott scattering for each group: $\Delta {E}_{Mott}={\sum }_{i=1}^{n}{\Delta E(\theta }_{i})$. The detail is to be discussed shortly.
8. Sum up each flying distance within each group to get the combined flying segments. The sum is expressed as ${\sum }_{i=1}^{n}{L}_{i}$,
9. Use the combined flying segments ${\sum }_{i=1}^{n}{L}_{i}$ and the continuous excitation/ionization energy loss to calculate other energy loss: $\Delta {E}_{non-Mott}=\left[{\left(\frac{dE}{ds}\right)}_{ionization}+{\left(\frac{dE}{ds}\right)}_{Brems.}\right]\times {\sum }_{i=1}^{n}{L}_{i}$.
10. Subtract the kinetic energy of the electron by both the energy loss from Mott scattering and the energy loss from continuous non-Mott scattering. The final energy, ${E}_{end (current)}$, is the new starting kinetic energy for the next group of flying distances: ${E}_{end (current)}={E}_{starting \left(current\right)}-\Delta {E}_{Mott}-\Delta {E}_{non-Mott}$.
11. Calculate the ratio of the midpoint energy to the starting energy of the current group, using $\alpha =\left[\left({E}_{starting (current)}+{E}_{end (current)}\right)/2\right]/{E}_{starting (current)}$.
12. Use the $\alpha$ value to estimate the midpoint energy of the next group via ${E}_{middle}(next)={E}_{end}(current)\times \alpha$.
13. Use the scattering angles from each Mott scattering within the current group to calculate the total accumulated deflection for the current grouped flying segments. The final scattering direction is the starting direction for the next group of flying distances. The calculation details will be discussed shortly.
14. Repeat the above cycles until the electrons either leave the substrate as backscattered electrons or stop inside the substrate. Stopping is determined based on whether the kinetic energy after the last collision is below a threshold energy for stopping, which is set to be 0.02 keV.

**Figure 6:**
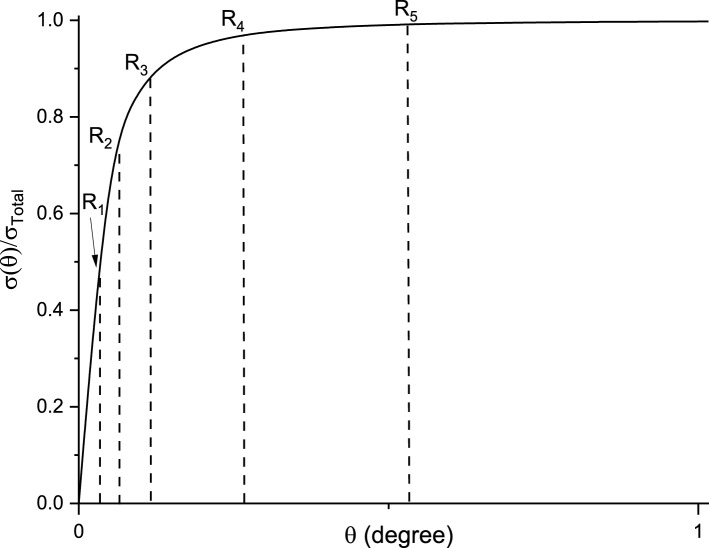
Obtaining θ_i_ values for each R_i_ for a group of five random numbers in ordered sequence from low to high.

Figure 6 further explains step 4. For simplicity, only five random numbers are shown. These five randomly produced values of *R* within the range (0,1) are sorted to satisfy the relationship that *R*_*1*_ < *R*_*2*_ < *R*_*3*_ < *R*_*4*_ < *R*_*5*_. During the integration of $\frac{d{\sigma }_{Mott}\left(\theta \right)}{d\Omega }$ to obtain the solid curve in Fig. 6, corresponding θ_i_ value for each *R*_*i*_ are obtained. Since *R*_*i*_ is in ascending, the integration process can identify all θ values at once. This is key to avoiding individual integration for each *R*. Afterwards, re-ordering of *R* within a group will be performed for the subsequent scattering simulations.

**Figure 7:**
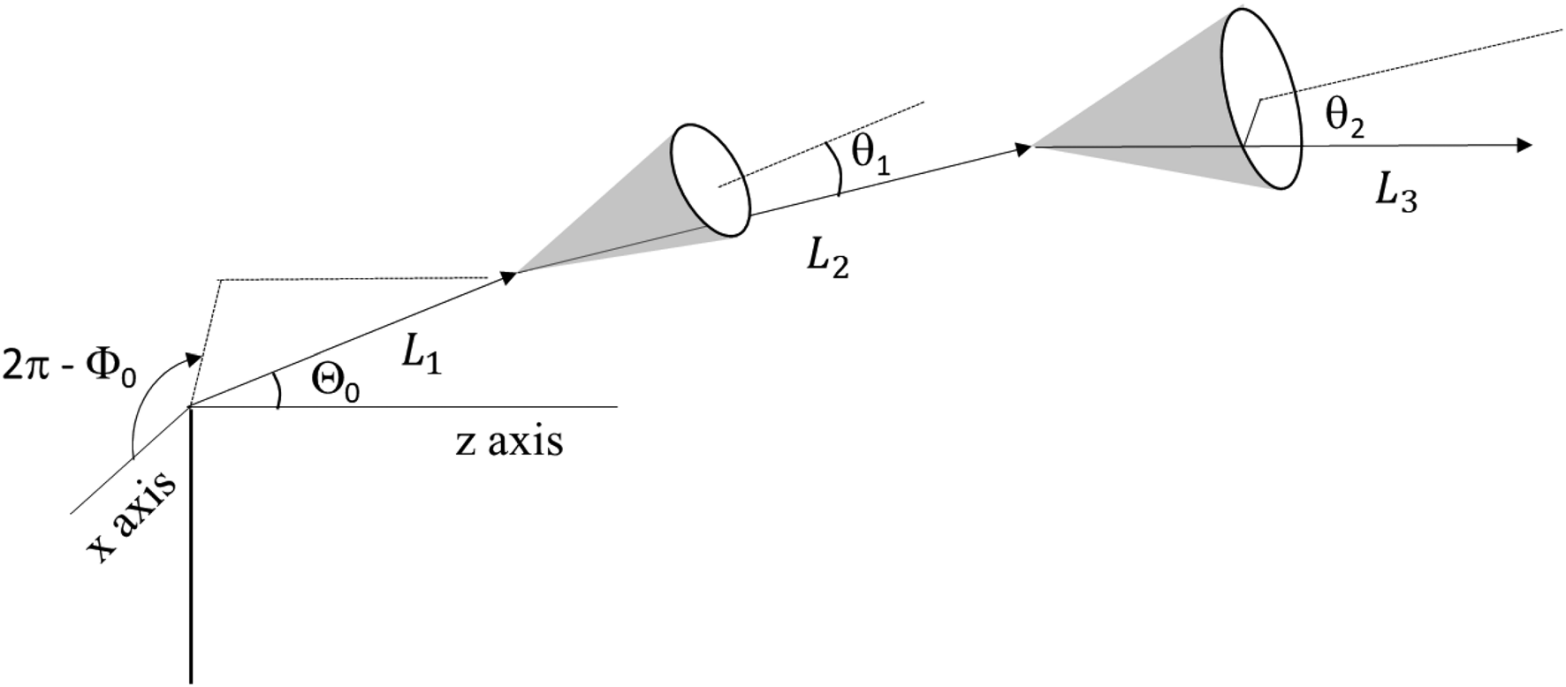
Schematics of angle definitions.

The schematics in Fig. 7 illustrate the definitions of angles. For step 13, after each Mott scattering within the group, the new directions, $\Theta$ with respect to the z-axis of the Cartesian coordinate, and Φ with respect to the angle away from the x–z plane of the coordinate, are obtained using the following conversion:

$$\sin \Theta \cos {\Phi } = cos{\Phi }_{0} cos\Theta_{0} cos\varphi_{i} sin\theta_{i} + cos{\Phi }_{0} sin\Theta_{0} cos\theta_{i} + sin{\Phi }_{0} sin\theta_{i} sin\varphi_{i}$$

$$\sin \Theta \sin {\Phi } = - sin{\Phi }_{0} cos\Theta_{0} cos\varphi_{i} sin\theta_{i} - sin{\Phi }_{0} sin\Theta_{0} cos\theta_{i} + cos{\Phi }_{0} sin\theta_{i} sin\varphi_{i}$$

20 $$\text{cos}\Theta =cos{\Theta }_{0}cos{\theta }_{i}-sin{\Phi }_{0}sin{\theta }_{i}cos{\varphi }_{i}$$

where ${\Phi }_{0}$ and ${\Theta }_{0}$ are the angles before scattering, and Φ and Θ are the new angles after scattering. ${\theta }_{i}$ is obtained from step 4. ${\varphi }_{i}$ is a value randomly assigned in the range of 0 to 2π, based on the assumption that the substrate is amorphous. The above angle conversions will be repeated *n* times for each group.

All energy losses are energy-dependent. The equal energy approximation within the same group requires an appropriate selection of electron energy. Using the energy midpoint of the group for scattering calculations is a straightforward choice. In the present study, an explicit method for such energy approximation is used, as described in steps 11 and 12. The energy midpoint of the next group is obtained using the final and starting energies of the current group. This method assumes that the ratio of the energy middle point to the starting energy of the current group is the same for the next group. The simulation of the next group yields energy information that allows a new ratio to be calculated for the subsequent group.

### Displacement production

The simulation easily integrates with calculations of various by-products of electron bombardment, such as displacement production. For that purpose, once the transferred energy to target atoms exceeds the threshold displacement energy, information about the primary knock-on, such as displacing direction and kinetic energy gained, is transferred to a subroutine. This subroutine is SRIM-like and capable of full damage cascade simulations. For the completion of the discussion, below the major steps involved in the full damage cascades are listed, and the results are shown in Fig. 10a-4, b-4, and c-4.

The ion–solid interaction subroutine has the following sequential steps for displacement calculations^29^:

(1) A random number is produced to find the impact parameter using the following equation

21 $$b={\left[\frac{R}{\pi {N}^\frac{2}{3}}\right]}^\frac{1}{2}$$

where *N* is the atomic density of the target atoms, *R* is a random number

(2) Once the collision parameter $b$ is obtained, it is used as input for the following equation to calculate the minimum separation distance between the knock-on atom and target atoms in the center of mass coordinate.

22 $$\frac{{b}^{2}}{{r}_{min}^{2}}+\frac{V\left({r}_{min}\right)}{{E}_{c}}-1=0$$

where the potential *V* is the ZBL potential^30^, which is widely used in ion-solid interaction community. It was obtained through fitting various ion-target systems, considering quantum mechanics effects. The ZBL potential is a binary potential that considers the electron screening effect between the ion and the target nucleus. This screening effect acts as a modification of the Coulombic interaction between ion and target nucleus. The ZBL potential is expressed as^30^:

23 $$V\left(r\right)=\frac{1}{4\pi {\varepsilon }_{0}}\frac{{Z}_{1}{Z}_{2}{e}^{2}}{r}\varphi \left(\frac{r}{a}\right)$$

where ${Z}_{1}$ and ${Z}_{2}$ are the atomic numbers of the knock-on and the target nuclei, respectively, and $r$ is the distance between the knock-on and target nuclei. The screening parameter $a$ is described as^31^:

24 $$a=\frac{0.8854{a}_{0}}{{Z}_{1}^{0.23}+{Z}_{2}^{0.23}}$$

where ${a}_{0}$ (${a}_{0}$=0.529 Angstrom) is the Bohr atomic radius. The screen function is calculated as:

25 $$\varphi \left(x\right)=0.1818{e}^{-3.2x}+0.5099{e}^{-0.942x}+0.2802{e}^{-0.4029x}+0.02817{e}^{-0.2016x}$$

(3) The curvature of the radius at the minimum separation distance in the center of mass coordinates is calculated using the following equation:

26 $$\rho =2\frac{\left[{E}_{c}-V\left({r}_{min}\right)\right]}{-{V}{\prime}\left({r}_{rmin}\right)}$$

(4) The scattering angle of the knock-on in the center of mass coordinates is calculated using Biersack's “magic formula”^29^:

27 $$\mathit{cos}\frac{{\theta }_{c}}{2}=\frac{B+{R}_{c}+\Delta }{\left({R}_{0}+{R}_{c}\right)}$$

where all parameters on the right are obtained from fitting and are calculable once ion-target systems and ion energies are known.

(5) The scattering angle is used to calculate the energy transfer to the target atoms, using the following equation^29^:

28 $$T=\frac{4E\cdot {m}_{1}\cdot {m}_{2}}{({m}_{1}+{m}_{2}{)}^{2}}{\mathit{si}n}^{2}\frac{{\theta }_{c}}{2}$$

where m_1_ and m_2_ are masses of the knock-on and target atoms, respectively. Transferred energy *T* will be subtracted from the Fe knock-on, accounting for nuclear stopping power loss. *T* is also used to judge whether additional target atoms will be displaced, depending on whether *T* is greater than the threshold displacement energy.

(6) The calculation is repeated for another flying distance, which is equal to the nearest neighboring distance. There is no need to use the free flying distance approach to save time, since the energy transfer from electrons to target atoms is very small. A 'monolayer' approach is sufficient and appropriate. The approach uses the average atomic distance of Fe as the flying distance between two scattering simulations.

(7) Before the next ion-target scattering simulation, the electronic energy loss is calculated. This energy, which contributes to electron excitation in Fe, is very small for low-energy ion bombardment and is treated as continuous energy loss. More details about electronic energy loss calculation can be found in reference^29^.

## Results and discussions

**Figure 8:**
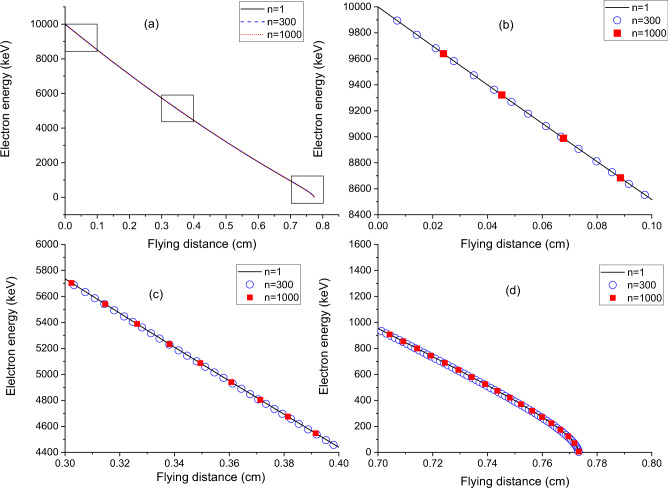
(**a**) Electron kinetic energy as a function of flying distances for three group numbers, n = 1, n = 300, and n = 1000. (**b**) Enlarged region near the surface, (**c**) enlarged region in the middle of the penetration, and (**d**) enlarged region at the end of their ranges. The number of angle intervals is 1000.

The proposed grouping of flying distances, even with *n* = 1000, does not cause noticeable errors in the energy loss of electrons along their flying paths. Figure 8a compares the electron kinetic energies for three individual bombardment events with *n* = 1, 300, and 1000. All three curves overlap well. Three boxes refer to the near-surface region, the middle, and the end of penetrations. For the near-surface region, as shown in Fig. 8b, all three curves exhibit the same amount of energy loss. The solid line, hollow circle, and solid square refer to n = 1, 300, and 1000, respectively. In the middle range regions, as shown in Fig. 8c, all three curves still follow each other closely. At the end of the projected ranges, as shown in Fig. 8d, they are still indistinguishable. This is a consequence of Mott scattering not being a significant energy loss mechanism. Hence, individual electron trajectories, even with large variations in Mott scattering details, do not bring noticeable differences to the total flying distance. If Mott scattering details are not able to differentiate one trajectory from another, it justifies our grouping method by ignoring the differences between neighboring flying distances and by using the approximated same energy within the group for scattering angle selection. Note that all three electrons stop their penetrations at the same distance when the energies are below 20 eV.

**Figure 9:**
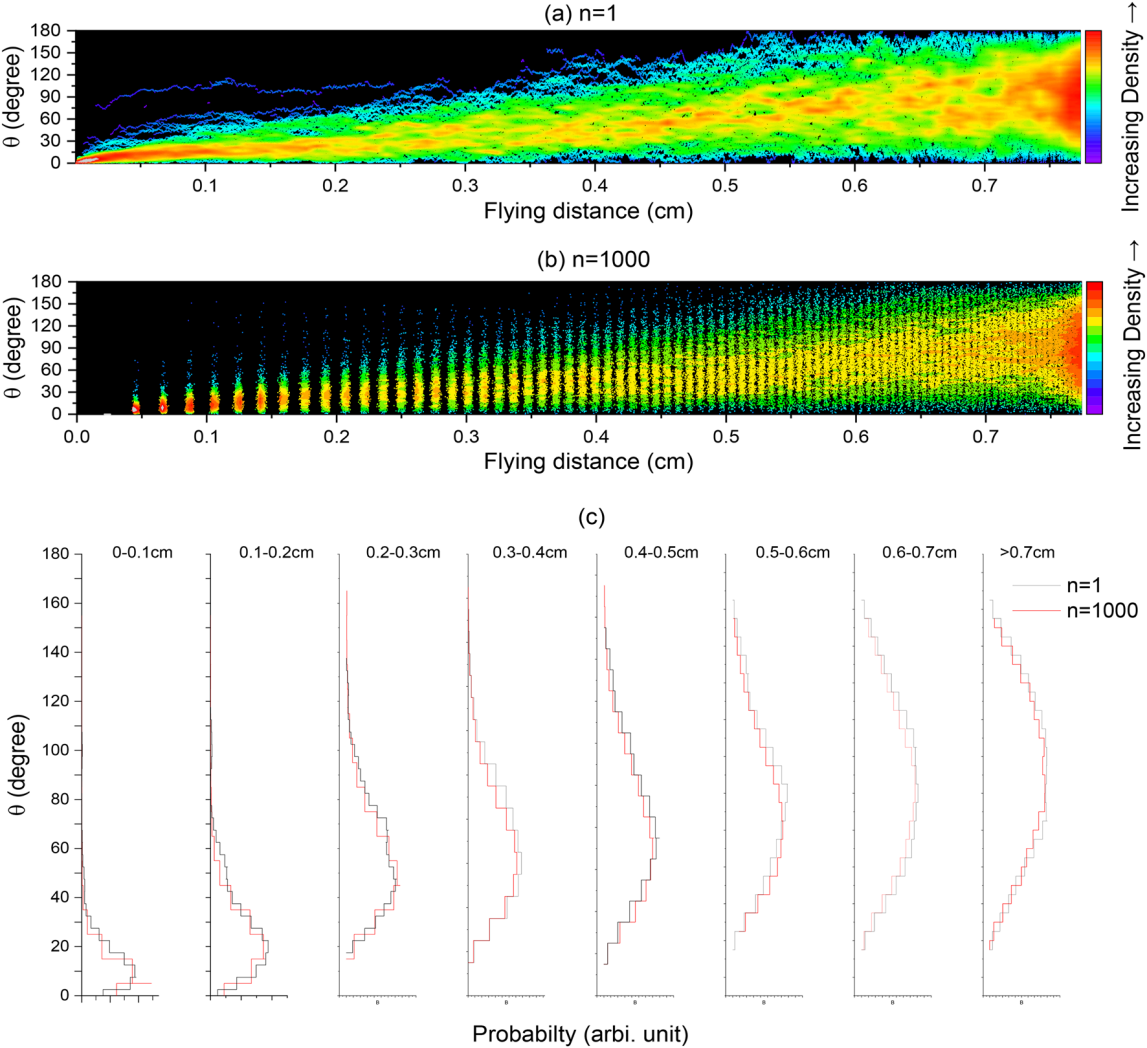
(**a**) Density plot of scattering angles for 1000 electron bombardments in Fe as a function of their flying distances, for the case of n = 1 (without L grouping), (**b**) density plot of scattering angles for 1000 electron bombardments for the case of n = 1000, (**c**) statistics analysis of scattering angles for both n = 1 and n = 1000 over eight distance regions of 0–0.1 cm, 0.1–0.2 cm, 0.2–0.3 cm, 0.3–0.4 cm, 0.4–0.5 cm, 0.5–0.6 cm, 0.6–0.7 cm, and > 0.7 cm, respectively. The number of angle intervals is 1000.

The grouping method does not cause noticeable errors in the scattering angle statistics distributions either. Figure 9a plots the θ distributions for 1000 electron bombardments as a function of their flying distance, for the case of *n* = 1 (no grouping). The color indicates the density of data points,  increasing from blue to red. θ values gradually increase from 0, as the initial bombarding conditions, to larger numbers with increasing penetration. At the very end of the penetration, θ becomes widely distributed over the whole 0 to π region. Figure 9b is a comparison with Fig. 9a, but for the case of n = 1000. Since flying distances are grouped, scattering analysis occurs after every 1000, which leads to a discrete pattern, instead of random distributions. At the very end, a similar spreading over 0 to π was observed. Since the free flying distance for each Mott scattering becomes smaller at low energies, the distance between neighboring groups becomes smaller, and the data density increases. The color mapping shows a similar density distribution at the very end. As a statistical summary, Fig. 9c compares scattering angle statistics distributions at eight distance intervals, each interval is 0.1 cm wide, except for the last one. The black solid line refers to *n* = 1 and the red solid line refers to *n* = 1000. At all depth regions, both *n* = 1 and *n* = 1000 show good agreement: the scattering angle peak shifts gradually to a larger number with increasing depth. Additionally, the distribution becomes broader. At the last distance interval, the scattering angle peaks at about 90 degrees with symmetrical distributions, indicating that about half of the electrons maintain forward directions and half maintain backward directions when they stop.

**Figure 10:**
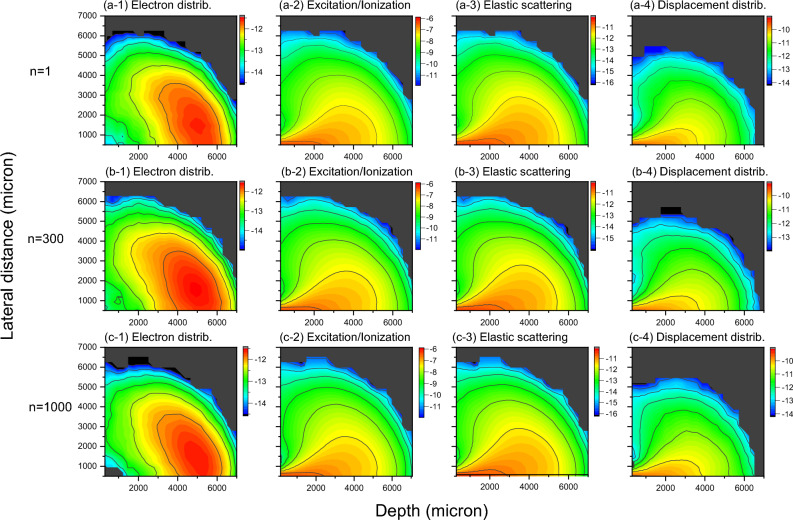
(**a**-1, **a**-2, **a**-3, **a**-4) 3D plots of electron distributions, excitation/ionization energy deposition, elastic scattering energy deposition, and target atom displacement production for 10 MeV electron bombardment in Fe, for n = 1 (no grouping of flying distances), (**b**-1, **b**-2, **b**-3, **b**-4) plots of the same but for n = 300, (**c**-1, **c**-2, **c**-3, **c**-4) plots of the same but for n = 1000. The color, from blue to red, indicates the value change from low to high. The color refers to the log scale of the corresponding values. For electron distributions, the value is the number of electrons per cm^3^ per incident electron. For inelastic and elastic energy deposition, the values are in the unit of keV per cm^3^ per incident electron. For the Fe displacement plot, the unit is the number of Fe displacements per cm^3^ per incident electron. All plots are obtained using 3000 electron bombardments. The number of angle intervals is 1000.

A comprehensive comparison is made in Fig. 10 to show the equivalence and accuracy of the *L* grouping method for the calculations of electron distributions, energy deposition, and displacement production. Figure 10a-1 is the plot of the electrons as a function of their radius and penetration depth in polar coordinates for the case of *n* = 1 (no grouping). We selected polar coordinates since the yield is symmetrical. In a comparison, similar 3D distribution patterns are obtained for *n* = 300 (as shown in Fig. 10b-1) and for *n* = 1000 (as shown in Fig. 10c-1). Both the pattern shape and peak location agree for the three cases. The patterns are characteristic of particles which experience backscattering. Figure 10a-2, 10b-2, and 10c-2 compare the deposition of ionization/excitation energy for *n* = 1, 300, and 1000, respectively. All three agree with each other, featuring the highest energy depositions at the very beginning of the bombardment. Figure 10a-3, 10b-3, and 10c-3 show energy deposition for elastic scattering (Mott scattering). All patterns agree with each other and are very similar to the pattern of inelastic scattering. As discussed already from Fig. 4, elastic scattering energy loss is more likely a systematic shift of inelastic scattering energy loss as a function of energy. Therefore, it is no surprise that both share similar patterns, but the amplitude of elastic scattering energy loss is systematically lower than that of inelastic scattering energy loss. Figure 10a-4, 10b-4, and 10c-4 compare the displacement creation of Fe atoms. The displacement production is a byproduct of Mott scattering, and occurs only when the transferred energy to target atoms exceeds the displacement threshold energy (40 eV for Fe). Hence, the displacement pattern is similar to that of elastic energy loss pattern but has a cutoff when the energy loss density is below a threshold, which leads to 'shrunken' patterns in comparison with elastic energy deposition patterns.

**Figure 11:**
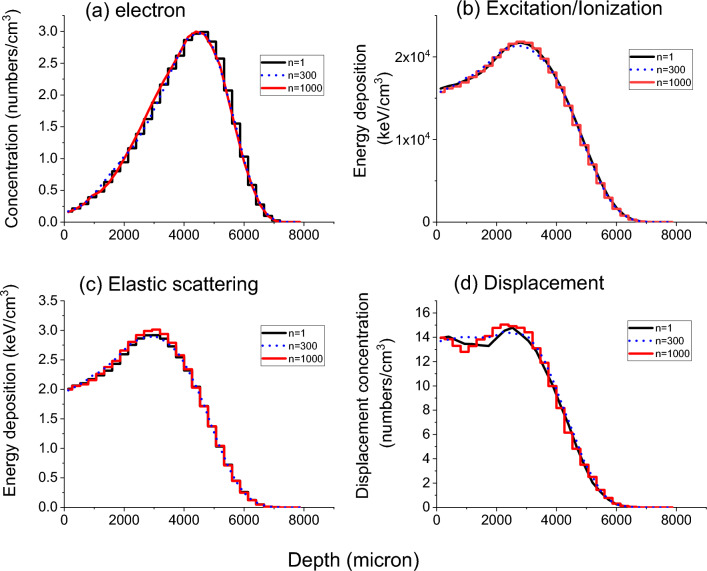
One-dimensional depth profiles of (**a**) electron distributions, (**b**) excitation/ionization energy deposition, (**c**) elastic scattering energy deposition, and (**d**) target atom displacement production for 10 MeV electron bombardment in Fe, for n = 1, 300, and 1000. All results are normalized to one electron.

Figure 11a–d compares the one-dimensional distributions of electron distributions, excitation/ionization energy deposition, elastic scattering energy deposition, and target atom displacement production electrons as a function of depth for 10 MeV electron bombardments in Fe. The profiles were obtained using the 3D profiles in Fig. 10. All these plots agree well for three cases: n = 1, 300, and 1000.

**Figure 12:**
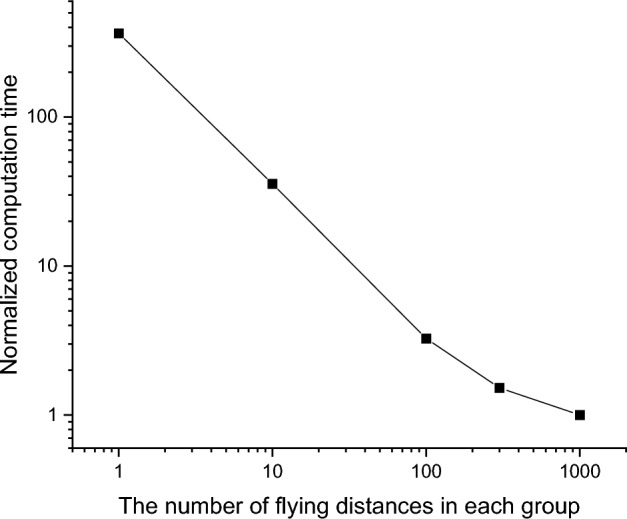
Computation time for simulating 10 MeV electron bombardment in Fe as a function of the number of flying distances grouped together in one group. The number of angle intervals is 1000.

Figure 12 summarizes the computation time required for the simulations, taking flying distance group sizes as *n* = 1, 10, 100, 300, and 1000. All times are normalized against that taken for n = 1000. There is a significant improvement in efficiency when n changes from 1 to 100. Efficiency appears to saturate at *n* ≥ 300. Comparing n = 1 with *n* = 1000, it is evident that the proposed method increases efficiency by approximately 400 times. All calculations were obtained using an angle interval number of 1000.

In the present study, the Mott differential scattering cross-section is calculated using R_Mott_ obtained from fitting^26^. The fitting formula with tabulated parameters is very helpful in reducing computation time and broadening the applicability for a wider energy region and more general target atoms. The error is less than 1.0% for atomic numbers Z = 1 to 44 for energies from 1 keV to 900 MeV.

**Figure 13:**
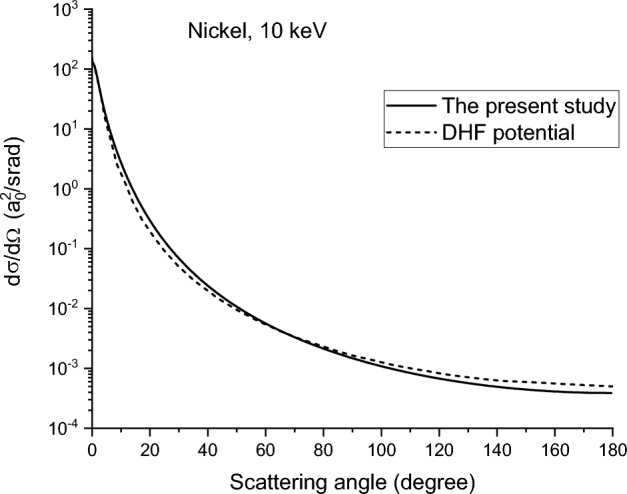
Comparison of the Mott scattering differential cross sections obtained from the present study and Reference^31^.

To assess the differences between the present study and the exact solutions from other groups, a comparison is made in Fig. 13. The solid line shows the results combining fitting-obtained R_Mott_ and the Dirac-Hartree–Fock-Slater screening function^26,27^, and the dashed line shows the exact solution calculated from the Dirac-Hartree–Fock potential for 10 keV electron irradiation in nickel^31^. The solid line is slightly higher than the dashed line at angles less than 70 degrees. For angles greater than 70 degrees, the dashed line becomes slightly higher than the solid line. The deviation of the two curves over the entire range is less than 40%. Figure 13 shows that for the most important scattering angle range, less than 5 degrees, the two curves are almost identical.

## Conclusion

We proposed a new method to speed up Monte Carlo simulation of electron-solid interaction which considers inelastic scattering, elastic scattering, and bremsstrahlung, for energy ranges up to 900 MeV. The key approach is to maximize the accuracy of integration of differential Mott scattering using small angle intervals and to minimize the number of integrations. The latter is a key limiting factor in computational speed. The method combines free flying distances into groups, with each group containing up to 1000 free flying distances. The scattering angle selections for each free flying distance within the group are achieved using only one integration process. The grouping method assumes that electron energy within each group is approximately constant. This assumption is supported by the fact that Mott scattering contributes an energy loss which is a few orders of magnitude lower than that from excitation and ionization. The grouping method does not affect the total flying distance nor the statistical distribution of scattering angles. For the extreme case of grouping 1000 neighboring free flying distance for each group, the computation speed increases by more than two orders of magnitude, while the obtained 3D distributions of electrons, energy depositions, and displacement creations still agree well with the simulation without grouping.

## Data Availability

The datasets generated during and/or analysed during the current study are available from the corresponding author on reasonable request.
